# High-Yield Alpha-Cellulose from Oil Palm Empty Fruit Bunches by Optimizing Thermochemical Delignification Processes for Use as Microcrystalline Cellulose

**DOI:** 10.1155/2023/9169431

**Published:** 2023-02-16

**Authors:** Susi Susi, Makhmudun Ainuri, Wagiman Wagiman, Mohammad Affan Fajar Falah

**Affiliations:** ^1^Department of Agroindustrial Technology, Faculty of Agriculture, Lambung Mangkurat University, Jl A Yani Km 36, Banjarbaru, South Kalimantan 70714, Indonesia; ^2^Doctoral Programme of Agroindustrial Technology, Faculty of Agricultural Technology, Gadjah Mada University, Jl Flora No. 1, Bulaksumur, Yogyakarta 55281, Indonesia; ^3^Department of Agroindustrial Technology, Faculty of Agricultural Technology, Gadjah Mada University, Jl Flora No. 1, Bulaksumur, Yogyakarta 55281, Indonesia

## Abstract

Oil palm empty fruit bunches (OPEFB) are lignocellulosic materials that are a by-product of the palm oil industry, which have less use and utilization is still limited. OPEFB's high cellulose content could potentially develop into various bioproducts, especially biomaterials. The thermochemical delignification process can obtain high-yieldalpha-cellulose. The cellulose extraction process can be done by combining the bleaching process under acidic conditions and alkaline delignification to obtain high-purity cellulose. The bleaching conditions vary in the concentration of NaClO_2_, the length of bleaching, the temperature, and the number of stages. The research obtains high *α*-cellulose by optimizing bleaching conditions under acidic conditions in cellulose's OPEFB extraction with variability on NaClO_2_ concentration and bleaching time using response surface methodology (RSM). The bleaching process was implemented at an early stage with a concentration of 3% NaClO_2_ and a bleaching time of 2 hours as a center point with a bleaching cycle of twice at pH 4–4.5 using acetic acid. Bleached fibers were delignified using 10% NaOH for 2 hours at room temperature. The RSM analysis resulted in optimum bleaching conditions at a concentration of 3.22% NaClO_2_ for 1 hour, yielding OPEFB's cellulose of 82.96% ± 2.53, hemicellulose of 9.27% ± 2.28, and lignin of 1.68% ± 0.58. The validation and verification process in the bleaching conditions obtained cellulose of 84.87% and *α*-cellulose of 88.51%, with a crystallinity index of 70.55% and crystallite size of 2.35 nm. Scanning electron microscopy on surface cellulose morphology at optimum bleaching helped remove hemicellulose impurities, lignin, and inorganic materials and a more intensive opening of cellulose fibrils. The bleaching process optimization point was verified to improve the delignification performance and potentially produce high yield *α*-cellulose content for microcrystalline cellulose use.

## 1. Introduction

Indonesia is the largest oil palm producer in the world and has the largest plantation. In 2021, Indonesia will have an area of 15 million hectares with a production of 49.71 million tons [1]. The potential for oil palm empty fruit bunches (OPEFB) waste is 25% (12.42 million tons) and utilized only 10%. OPEFB waste potential is 15 percent or 11.18 million tons [2]. OPEFB is relatively abundant [3] and has benefits as an alternative energy source [4, 5], fertilizers, chemical components, and biomaterials [6].

Previous research reported that OPEFB contained cellulose in amounts of 41.8% [7], 37.6% [8], 32.80% [9], 36.2% [10], and 34.83% [11]. OPEFB has an high cellulose content, making it an auspicious raw material for conversion into nanofiber [12], microcrystalline cellulose [13–15], nanocrystalline cellulose [16], and the production of various cellulose-based products.

OPEFB, as the biomaterial source, needs proper cellulose isolation technology to obtain high purity so it can be optimized using cellulose as its derivative product. The potential market for microcrystalline cellulose products is quite large in the pharmaceutical, cosmetic, and food industries. Microcrystalline cellulose is widely used as a binder [17–20], filler [21–26], and absorbent [27, 28]. OPEFB cellulose, as a raw material for microcrystalline cellulose, requires high levels of alpha cellulose, which affects its crystallinity. OPEFB has a complex and waxy structure; therefore, an efficient extraction process is required. Chemical treatment is the most effective way to obtain higher-purity cellulose. Combining bleaching with NaClO_2_, alkali treatment, and acid hydrolysis is commonly used for extracting cellulose [29–32].

Chemical methods using alkaline and acid solutions help remove lignin and some hemicelluloses and reduce the degree of polymerization. This technique requires a low cost to remove acetyl groups and lignin at low pressure and low temperature and increases the crystallinity of cellulose [16, 33, 34].

Several methods have been studied. Removing hemicellulose and lignin fractions in physical hydrothermal pretreatment was carried out at a temperature of 150°C to 200°C at a high pressure of about 30 bar with a reaction time of 5–25 minutes [35]. This method is environmentally friendly but requires high energy. Pretreatment using an ionic solution such as bmimHSO_4_ (1-butyl-3-methylimidazolium hydrogen sulfate) has a high solvation but is expensive [36]. Ultrasound-H_2_O_2_ techniques at low temperatures and shorter times are not optimal in cellulose isolation [37]. Meanwhile, steam explosion treatment at 160–260°C in a short time can reduce lignin and hemicellulose content but can cause excessive degradation of cellulose. The enzymatic delignification method is environmentally friendly but results in slow delignification [38].

In the long term, the combination of sodium chlorite and alkaline pretreatment of OPEFB is an economical method for pulp treatment due to the application of low operating temperatures and pressures [33, 34]. The preparation conditions, such as the type of chemicals used, the concentration used, and the duration and temperature of the hydrolysis treatment, influenced the yield of cellulose extracted from the fiber. Previous studies performed cellulose extraction from plants using alkaline treatment, followed by NaClO_2_ bleaching [39–43]. Alkali treatment was only able to partially remove hemicellulose and lignin. Chemical treatment, such as acidic bleaching, can improve high cellulose content. Therefore, the bleaching process obtains the desired purity of cellulose.

Bleaching before alkaline delignification using 0.7% NaClO_2_ for five cycles resulted in 81.1% cellulose [15]. Pujiasih et al. [14] prepared OPEFB cellulose to produce MCC with a crystallinity index of 66.99% through bleaching of 15% NaClO_2_ for three cycles and a long OPEFB cellulose preparation step to find extracted cellulose for CMC production [44]. Based on preliminary research results, delignification using 17.5% NaOH at room temperature and bleaching under acidic conditions using NaClO_2_ 3% twice produced cellulose greater than 80%. Under acidic conditions, the bleaching process not only plays a role in whitening the fiber but also helps extract cellulose more optimally.

Chemical processes effectively produce high-purity cellulose. The delignification process cannot work alone but requires a combination of bleaching under acidic conditions. The delignification process with NaOH and KOH was only partially able to remove lignin and hemicellulose. The isolation process was complemented by a bleaching process with NaClO_2_ under acidic conditions to remove lignin, hemicellulose, and partial depolymerization.

An efficient process requires a low temperature, a low bleacher concentration, and a short bleaching cycle, thereby shortening the production process. Optimizing the bleaching process under acidic conditions to help remove lignin and hemicellulose has not been investigated. The acidic conditions in the bleaching process are adjusted using acetic acid, which is environmentally friendly. The acidic conditions for bleaching are carried out after reaching 70°C so that NaClO_2_ has reacted effectively. In previous studies, the acidic conditions of bleaching were not clearly explained. Therefore, this research aimed to optimize the bleaching under acidic conditions, especially to get the optimum bleacher concentration and bleaching time for process efficiency and enhance delignification that produces high-content*α*-cellulose. Obtaining optimization of bleacher concentration, minimum cycle, and minimum processing time with a moderate process will be very beneficial for the industrialization of *α*-cellulose from OPEFB in the oil palm industry. The conversion of OPEFB waste into derivative products can improve its added value.

## 2. Materials and Methods

### 2.1. Materials

Oil palm empty fruit bunches were supplied from PT Nurciptasari Moeda Sentosa South Kalimantan, NaOH (Merck), NaClO_2_ (Clover Chemicals Ltd), glacial acetic acid (Merck), H_2_SO_4_ (Merck), and Aquadest. The equipment used includes a glass beaker, a hotplate stirrer, a spatula, filter paper, filter cloth, attenuated total reflectance-Fourier transform infrared spectroscopy (ATR-FTIR) (Bruker 200546 Model Alpha, Australia), X-ray diffraction (X-RD) (Rigaku MiniFlex 600, Japan), and scanning electron microscopy (SEM) (Brand FEI Inspect-S50 type, Japan).

### 2.2. Experimental Design from Response Surface Methodology

The primary data on the chemical quality of cellulose were collected from each treatment, which included water extractives, hemicellulose, cellulose, and lignin. The treatment was designed from an optimization process carried out using the Response Surface Methodology (RSM) with two factors, namely, the bleaching time with a center point of 2 hours and NaClO_2_ concentration with a center point of 3%, thus obtaining 13 experimental units with 5 points as repetitions at the center. The design used for the optimization analysis uses a central composite with a quadratic target model; the details of the factors are in Table 1 and the experimental design is carried out according to Table 2.

### 2.3. OPEFB Preparation

OPEFB was washed with hot water, and the fibers were separated manually. Fibers were rinsed for up to quartz washings using clean water. Furthermore, the fiber was soaked in a 2% soap solution (ratio of fiber to soap = 1 : 4) for 5 hours to remove residual oil and dust. The remaining soap (and other dirts) was rinsed with clean water twice. The washed fibers were then drained and dried in an oven at 60°C for 48 hours. The clean and dry OPEFB was cut into ±5 cm, ground, and then sieved to obtain a size of 30 mesh.

### 2.4. Cellulose Extraction Process (Bleaching and Delignification)

Ten grams of OPEFB fiber was bleached using NaClO_2_ according to the treatment of NaClO_2_ concentration, with a fiber-to-NaClO_2_ solution ratio1 : 25 (w/v). The solution was heated until the temperature of 75°C ± 5°. Next, the bleacher solution was added acetic acid to adjust the pH to 4–4.5 and heated constantly at a temperature of 75°C ± 5° with bleaching time according to the treatment variation. Acidification by glacial acetic acid at pH 4–4.5 was done twice. After the first bleaching, the solution was filtered and continued in the second cycle at the same bleacher concentration and bleaching time. The bleached fibers were washed, dried, and weighed. The delignification of bleached fibers used a 10% NaOH solution with a ratio of 1 : 20 w/v at room temperature. The delignified cellulose was washed with hot, distilled water. The cellulose was refluxed for 30 minutes with distilled water for washing and then dried at 60°C ± 0.5° for 24 hours.

### 2.5. Characterization of OPEFB Fiber and Extracted OPEFB Cellulose

Cellulose was tested, including water-extractive material, hemicellulose, cellulose, and lignin. These parameters were used to optimize the process conditions. The chemical structure of cellulose was analyzed by attenuated total reflectance-Fourier transform infrared spectroscopy (ATR-FTIR) (Bruker 200546 Model Alpha), the crystallinity analysis was carried out by X-ray diffraction (X-RD) (Rigaku MiniFlex Hypix-400MF 2D HPAD detector),and the morphology analysis of OPEFB cellulose was carried out using scanning electron microscopy (SEM; Brand FEI, Inspect-S50 type).

### 2.6. Test Method for Fiber and Cellulose Components

Fiber components were analyzed using the Chesson method [45]. One gram of powder was added to 150 mL of distilled water and refluxed for 2 hours. The sample was filtered and washed until the pH was neutral, put in an oven at 105°C to dry, then weighed and calculated using equation (1). Residue 1 was added to 150 mL of 0.5 M H_2_SO_4_ and refluxed for 2 hours. The sample was filtered and washed until the pH was neutral, put in an oven at 105°C to dry, then weighed and calculated using equation (2). Residue 2 was added to 10 mL of 72% H_2_SO_4_ and macerated for 4 hours at room temperature. The sample was added to 150 mL of 0.5 M H_2_SO_4_ and refluxed for 2 hours. The sample was filtered and washed until the pH was neutral, put in an oven at 105°C to dry, then weighed and calculated using equation (3). The calculation of lignin content using equation (4) is as follows:

(1) $$\begin{array}{c} water\;extractive\;material\;content\left(\%\right)=\frac{initial\;mass-mass\;of\;residue\;1}{initial\;mass}\times 100\%\text{,} \end{array}$$

(2) $$\begin{array}{c} hemicellulose\;content\left(\%\right)=\frac{mass\;of\;residue\;1-mass\;of\;residue\;2}{initial\;mass}\times 100\%\text{,} \end{array}$$

(3) $$\begin{array}{c} cellulose\;content\left(\%\right)=\frac{mass\;of\;residue\;2-mass\;of\;residue\;3}{initial\;mass}\times 100\%\text{,} \end{array}$$

(4) $$\begin{array}{c} lignin\;content\left(\%\right)=\frac{mass\;of\;residue\;4}{initial\;mass}\times 100\%\text{.} \end{array}$$

### 2.7. X-Ray Diffraction

X-ray diffraction (XRD) was carried out to study sample crystallinity. The sample patterns of all cellulose samples were pressed and recorded by diffractometer using Ni-filtered Cu K*α* radiation (30 kV and 30 mA). Diffraction intensity was measured between the Bragg angle (2*θ*) of 3°–90°, with a scan speed of 10 degrees/min and a step width of 0.02 deg. The crystallinity index (CrI) was calculated by the Segal formula [46] using intensity measurements at 22.0°–23.0° and 15.0°–17.0° (amorphous) 2*θ*

(5) $$\begin{array}{c} CI\left(\%\right)=\left(\frac{I_{002}-I_{am}}{I_{002}}\right)\times 100\%\text{,} \end{array}$$

where *I*_002_ indicates the maximum intensity of the 002 peaks around 2*θ* = 22.0°–23.0° and *I*_*am*_ is the lowest intensity corresponding to the value of 2*θ* around 15.0°–17.0°

### 2.8. Data Analysis

Water extractive materials, hemicellulose, cellulose, and lignin data were analyzed using Response Surface Methodology with Design Expert version 12 software From StateEase, Minneapolis, to determine the optimization point on treatment factors. RSM tests the fit of the model regression (lack of fit), the regression parameters simultaneously, and the residual assumption that the residual must meet the normal assumption. Furthermore, a response surface analysis was carried out to obtain the optimum point. The data on the crystallinity index, crystallite size, and morphology of the cellulose structure were presented descriptively.

## 3. Results and Discussion

### 3.1. Cellulose, Hemicellulose, and Lignin

The content of hemicellulose, cellulose, and lignin in fibrous material varies greatly depending on the source of the material. The OPEFB fiber contains water-extractive material of 15.73%, hemicellulose of 31.51%, cellulose of 32.97%, and lignin of 19.79%. The results are comparable to other studies. The cellulose content in OPEFB ranges from 23.7 to 65.0%, hemicellulose is 20.58–33.52%, lignin is 14.1–30.45%, and water extractive material is 3.21–3.70% [47].

To obtain cellulose from OPEFB fiber, bleaching and delignification were combined. Extraction optimization was applied to the bleaching process of OPEFB using NaClO_2_ at pH 4–4.5 with two cycles of bleaching and delignification using 10% NaOH once. Optimal conditions are as follows: center point concentration of 3% and the length of a bleaching process is 2 hours.

The bleaching process tends to identify the decolorization process. However, the bleaching conditions at an acidic pH will help open the structure and partially depolymerize it, and reducing hemicellulose and lignin will be easier. The decrease in hemicellulose and lignin will continue at the base delignification stage.

The content of hemicellulose, cellulose, and lignin varies depending on the NaClO_2_ concentration and bleaching time. At the same bleaching cycle, which is twice, the concentration of NaClO_2_ and bleaching time will affect the cellulose and lignin content. A NaClO_2_ concentration of less than 3% is insufficient to produce high cellulose purity (>80%).  Table 3 presents data on the content of water extractive material, hemicellulose, cellulose, holocellulose, and lignin in extracted cellulose from OPEFB.

The cellulose obtained from the treatment varied depending on the concentration of NaClO_2_ and the bleaching time applied. A concentration of 3% NaClO_2_ with a bleaching time of 0.59 hours obtained the highest cellulose content. Lignin residues were still quite significant in the cellulose obtained in the C1.5T1, C0.88T2, and C1.5T3 treatments. During the same bleaching cycle, low NaClO_2_ concentrations are insufficient to reduce lignin in OPEFB fibers significantly. The NaClO_2_ concentration range of 3% to 5.12% significantly reduced lignin, and the residual lignin was 1–2.83%.

Bleaching under acidic conditions helps open the OPEFB cell wall structure, especially the outer layer, in the form of a wax layer. Bleaching using NaClO_2_ under acidic conditions has been used for cellulose isolation [14, 15, 44, 48, 49]. Research by Soetaredjo et al. [50] produced 77.8% cellulose from OPEFB with a combination of delignification of NaOH 2 N at 6 hours and microwave. Septevani et al. [8], using 10% NaOH delignification at 150°C pressure for 4 bar for 30 minutes and bleaching NaClO_2,_ were able to obtain 84.3% cellulose purity, and Yimlamai et al. [51] obtained 83.7% cellulose using peracetic acid in two stages and combinations of H_2_O_2_ and NaOH in the delignification process.

The intensive depolymerization process at the initial acid bleaching stage helps reduce the cellulose's impurities, hemicellulose, and lignin components. According to Mussatto et al. [52], acid pretreatment will disintegrate the fiber to facilitate delignification. Base components will quickly enter the structure, and the degradation of lignin is more efficient so that the release of cellulose occurs. Firstly, hydrolyzation quickly occurs in hemicellulose, causing hemicellulose to bind to cellulose with hydrogen bonds. Lignin binds to cellulose with covalent bonds on the inside of the cell structure, so that the acidic conditions in bleaching not only remove color components (chromophores) but also help remove lignin.

### 3.2. Determination of Model Optimization

The extraction of cellulose is optimized from OPEFB to obtain the optimum concentration of NaClO_2_ and bleaching time, which is capable of getting high purity of cellulose. Optimization was carried out on the water-extractive material, hemicellulose, cellulose, and lignin contents. There are several optimization parameters to consider sequentially, including fit summary, lack-of-fit tests, analysis of variance (ANOVA), and analysis of diagnostic plots for model validation, followed by analysis of multiple response optimization using graphical and numerical tools.  Table 4 presents a model that fits each parameter.

The fit summary showed the feasibility of the formed model from each response or parameter for water, hemicellulose, cellulose, and lignin extractive materials. The water-extractive material response was linear, with hemicellulose in 2F1 and cellulose and lignin in a quadratic model. The results of the ANOVA analysis for the response of water extractives and hemicellulose showed that the independent variables of bleaching time and NaClO_2_ concentration did not significantly affect the model (*p* value >0.05). Otherwise, the variable concentration of NaClO_2_ and the square of the concentration of NaClO_2_ significantly affect the model (*p* value <0.05) for cellulose and lignin responses.

The parameters of cellulose and lignin produced a quadratic model. In contrast, the air extractive material parameters were linear, and hemicellulose models were produced in the 2F1 model (between linear and quadratic). The lack of fit indicates the model's acceptance of the model, which is not significant (*p* value >0.05).  Table 5 shows the lack of fit value was insignificant for all parameters (*p* value >0.05), namely, the air extractive material parameter with a *p* value of 0.9948, a *p* value of hemicellulose 0.0998, a *p* value of cellulose 0.3721, and a *p* value for lignin 0.2024. Lack of conformity is a deviation or inaccuracy to the model, with an insignificant *p* value indicating the model is acceptable, and the error does not affect the model significantly.

**Figure 1:**
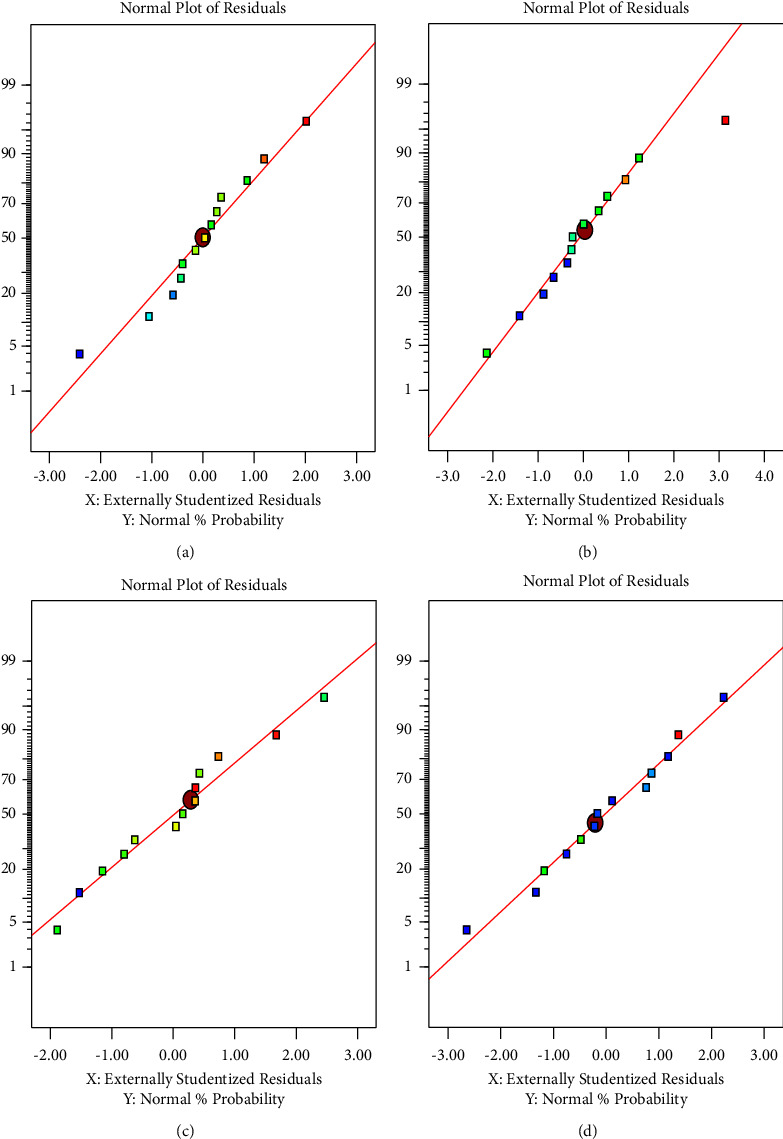
Normal plot response optimization of cellulose components: (a) water-extractive material, (b) hemicellulose, (c) cellulose, and (d) lignin.

The normality of the data must support the significance of the model. Data analysis showed that plotting residual data on the parameters of the extractive material water, hemicellulose, cellulose, and lignin is normal. The scattering of the data indicates a normal distribution following a straight line.  Figure 1 describes the normality data for all optimization parameters.

The model predicted a relationship with the response between the bleaching time (*X*_1_) and NaClO_2_ (*X*_2_) concentration. The significance model in optimization is described by the quadratic model. The responses of cellulose and lignin resulted in the quadratic model. The following is the equation of the model on the parameters of the water extractives (Ye), hemicellulose (Yh), cellulose (Ys), and lignin (Yg):

(6) $$\begin{array}{c} Ye=7.55+0.58X_{1}+0.2\text{,}\\ Yh=10.51+1.36X_{1}-0.76X_{2}-1.62X_{1}X_{2}\text{,}\\ Ys=80.32-2.06X_{1}+2.92X_{2}+1.78X_{1}X_{2}+0.47X_{12}-2.93X_{22}\text{,}\\ Yg=2.37+0.12X_{1}-2.43X_{2}-0.2427X_{1}X_{2}-0.28X_{12}+1.51X_{22}\text{.} \end{array}$$

**Figure 2:**
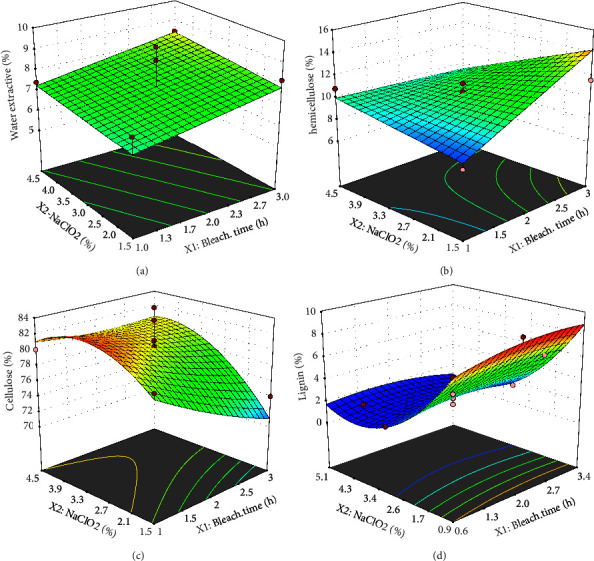
Contour and response surface model of (a) water-extractive materials, (b) hemicellulose, (c) cellulose, and (d) lignin.

The quadratic equation on the cellulose response (Ys) showed a positive correlation between the single factor of bleaching time and NaClO_2_ concentration, their interaction, and the square of the bleaching time factor (increases cellulose). However, the square of the concentration of NaClO_2_ has a negative correlation with the cellulose produced. In the quadratic model of the response of cellulose and lignin, the single-factor NaClO_2_ concentration and the squared concentration of NaClO_2_ had a significant effect on the model. In the quadratic equation of lignin response, the square of NaClO_2_ concentration positively correlated to lignin. However, the single factor of NaClO_2_ concentration, the interaction, and the square of the bleaching time factor had a negative effect (reduced) on the lignin content.  Figure 2 shows the contour and surface model responses for each response.

#### 3.2.1. Solution of Optimization

**Figure 3:**
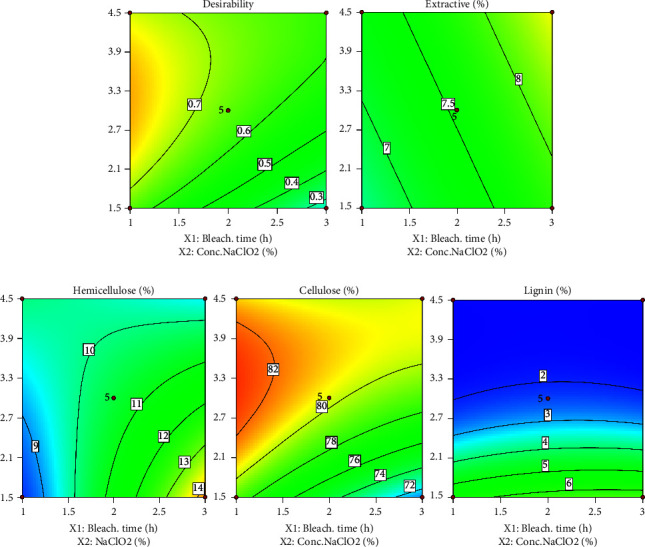
Point of optimization of process conditions and their parameters.

The optimal solution point for bleaching time and NaClO_2_ concentration was determined using the target parameter value approach. The constraints data to find the optimized point are presented in Table 6. Optimization conditions are justified in the water extractive material, hemicellulose, and lignin content at a minimum, while cellulose is at a maximum. Furthermore, all parameters were set at the same level of importance: level 3. The optimized bleaching process conditions determined by RSM resulted in optimized bleaching at a NaClO_2_ concentration of 3.22% and a bleaching time of 1 hour.  Figure 3 describes the validation of the optimization conditions. A quadratic model directs the optimization point on cellulose and lignin with maximum and minimum cellulose content.

#### 3.2.2. Optimization of Point Confirmation

The RSM found the optimization point of the bleaching process at a NaClO_2_ concentration of 3.22% and a bleaching time of 1 hour, and then the confirmation step was carried out. On the confirmed response surface at the optimization point, the cellulose content obtained was 82.96% ± 2.53 with a 95% confidence level.  Table 7 presents the complete data on confirmation from optimized bleaching. OPEFB cellulose obtained from the optimization point is higher than the standard cellulose content of 80.80% [8].

Confirmation and verification of optimization conditions were done by carrying out the bleaching process at 3.22% NaClO_2_ and a 1 hour bleaching time. After delignification, a cellulose content of 84.87%, *α*-cellulose 88.51%, hemicellulose of 9.80%, holocellulose of 94.67%, and water extractive material of 3.36%. The optimization point is verified to produce an optimal extraction process. Optimized bleaching conditions in acidic conditions before delignification can improve the performance of delignification and produce high-purity cellulose.

According to Septevani et al. [8], the alkaline delignification process combined with bleaching NaClO_2_ provides high selectivity, reducing hemicellulose and lignin simultaneously without damaging the cellulose structure. The average content of *α*-cellulose in the C3T2 and C3.22T1, respectively, was 84.52% and 88.51%. *α*-Cellulose in C3.22T1 treatment range from 83.02% to 94.00%. *α*-Cellulose is insoluble cellulose at a concentration of 17.5% NaOH. In this study, high *α*-cellulose is essential to produce microcrystalline cellulose (MCC) as raw material for hydrogel filler and enhance the hydrogel's mechanical strength. *α*-Cellulose has a high degree of polymerization. MCC is produced from partially depolymerized *α*-cellulose by hydrolysis of excess mineral acid [17]. MCC is characterized by a high degree of crystallinity, the value of which is usually in the range of 55% to 80% [53].

Kim [54] stated that cellulose is the main constituent of lignocellulosic biomass. The cellulose content of lignocellulosic biomass varies from 30 to 50%. Cellulose molecules combine as microfibers, in which highly ordered (crystalline) regions alternate with less regular ones (amorphous). Moreover, cellulose strongly tends to form intra and intermolecular hydrogen bonds. Hemicellulose has a less stable structure; that is, it is more amorphous than cellulose and consequently more easily hydrolyzed by acids into monomers.

Acid pretreatment conditions easily degrade into decomposition products, including furfural. The lignin molecules are cross-linked and have high molecular weights, ranging from 12% to 33% by weight in lignocellulosic biomass. Lignin functions in plants to unite cellulose fibers and provide strength to lignocellulosic biocomposites [55].

### 3.3. Fourier Transmittance Infrared Spectroscopy

FTIR analysis showed the consistency of the functional groups in cellulose. Bleaching treatment with various concentrations of NaClO_2_ and bleaching time will provide several changes, namely, a decrease in hemicellulose and lignin and an increase in the amount of cellulose that varies. The results of the FTIR analysis provide a qualitative description of the wave numbers on the structure of hemicellulose, cellulose, and lignin.

**Figure 4:**
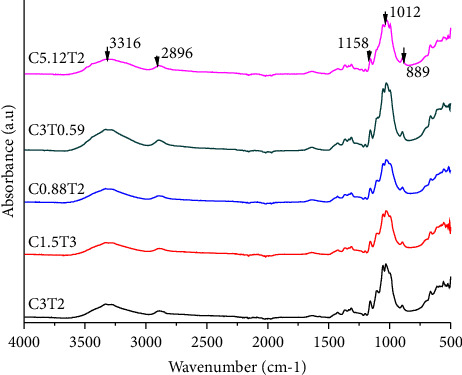
FTIR analysis on cellulose with various treatments of NaClO_2_ concentration and bleaching time (C concentration of NaClO_2_ (%); T = bleaching time (h)).

Figure 4 describes the dominant wavenumbers showing changes in cellulose, hemicellulose, and lignin at 3316 cm^−1^ (O-H stretching on cellulose, hemicellulose, and lignin), 2896 cm^−1^ (C-H stretching on cellulose, hemicellulose, and lignin), 1158 cm^−1^ (C-O-C asymmetric stretching in cellulose, hemicellulose), 1012 cm^−1^ (holocellulose and lignin, C-O stretching), and 889 cm^−1^ (C1-H deformation in cellulose) [56].

**Figure 5:**
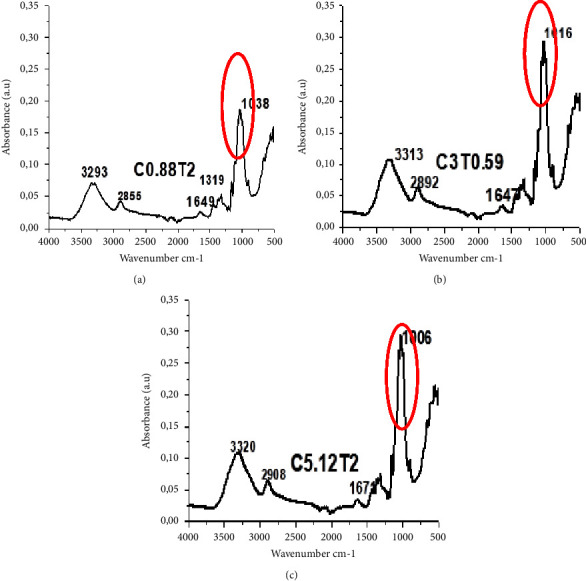
Comparison of wavenumbers in treatment: (a) C0.88T2, (b) C3T0.59, and (c) C5.12T2.

Figure 5 shows that the treatment of C3T0.59 and C5.12T2 sharpened the wave numbers in the range of 3300 cm^−1^, 2890 cm^−1^, and 1016 cm^−1^. The wavenumber is the area of cellulose and holocellulose. Shifting wavenumber indicated increased cellulose purity and decreased hemicellulose and lignin impurities. Popescu et al. [56] stated that changes in wavenumber could indicate cellulose structure degradation by acid-bleaching, and cellulose might be eroded and defibrillated.

Identification of wave numbers in the treatment C0.88T2, C3T0.59, and C5.12T2 as follows: at wavenumbers, 3300 cm^−1^ to 2900 cm^−1^ is a distinct area of O-H and C-H bonds in polysaccharides. The broad peak at 3320 cm^−1^ is the hydroxyl stretching vibration and the cellulose inter and intramolecular hydrogen bond vibrations. Typical wavenumbers indicated cellulose range from 1670 cm^−1^ to 900 cm^−1^. The absorbance peak of 1016 cm^−1^ correlates with the vibrations of the water molecules absorbed in the cellulose [57]. Bands at absorption 1649 cm^−1^, 1319 cm^−1^, and 1016 cm^−1^ produced stretching and bending vibrations of -CH2 and -CH, -OH, and C-O bonds in cellulose [58]. According to Zhang et al. [59], the chloric acid solution has been successfully used to break the ether bonds between lignin and cellulose; this is evidenced by the loss of aromatic skeletal vibrations in lignin at wavenumbers 1.505 cm^−1^ and 1.592 cm^−1^, and the ester bond between hydroxyl lignin and carboxyl uronic acid in hemicellulose is disrupted during alkaline treatment [16].

### 3.4. X-Ray Diffraction

An X-ray diffraction test showed changes in the crystallinity of cellulose by treatment with variations in NaClO_2_ concentration and bleaching time. The crystallinity index (CI) parameter describes cellulose's relative amount of crystalline material. The two-phase cellulose model describes the cellulose chains as containing crystalline and amorphous regions.

**Figure 6:**
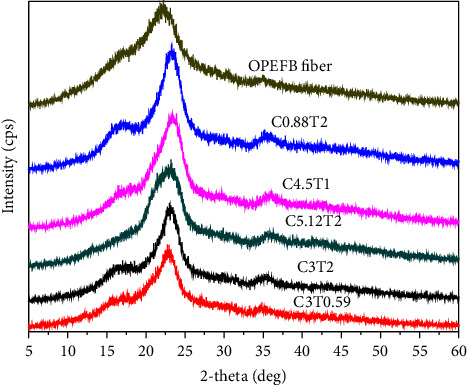
XRD analysis on cellulose with various concentrations of NaClO_2_ and bleaching time (C concentration of NaClO_2_ (%); T = bleaching time (h)).

XRD analysis found cellulose peak intensity at 2*θ* of 22°–23° and 2*θ* at the amorphous intensity at 15°–17°.  Figure 6 shows the peak sharpening in the C3T2 and C3T0.59 treatments compared to EFB fiber. In Table 8, the crystallinities of C3T2 and C3T0.59 were 85.78% and 86.56%, respectively. The highest crystallinity index in the C5.12T2 treatment was 89.15%. The cellulose crystallinity is higher than that of EFB fiber. Bleaching under acidic conditions and alkaline delignification can reduce hemicellulose and lignin, which have an amorphous structure. Kim [54] stated that the crystallite structure is related to the reduced amorphous structure.

Bleaching under acidic conditions followed by delignification degrades the amorphous cellulose structure, thereby increasing the degree of crystallinity. High crystallinity is very important in microcrystalline cellulose, especially in its use as a filler that can strengthen the mechanical structure, such as in tablets and hydrogel films.

Table 8 provides an overview of the consistent pattern of the effect of NaClO_2_ concentration and bleaching time. However, it does not fully describe a linear relationship where a higher NaClO_2_ concentration and bleaching time will increase the crystallinity index. The C4.5T3 treatment has a crystallinity index of 72.64%. This is in line with previous research. EFB fibers were bleached using 15% Na hypochlorous acid (1 : 25 w/v) for 2 hours at 80°C three times, followed by delignification using 17.5% NaOH (1 : 12.5 w/v) for 2 hours at room temperature twice to produce cellulose crystallinity of 66.99% [14]. Cellulose crystallinity is associated with tensile strength, which is essential to consider the cellulose used as its derivative product.

One of the causes of peak widening is the presence of an amorphous structure. However, on the other hand, intrinsic factors that affect peak widening include crystal size and nonuniform strain in the crystal. The peak in OPEFB is broader than in cellulose. The hemicellulose content is still high in OPEFB. Peak cellulose is due to amorphous cellulose. However, crystal size is equally important for peak broadening, and several studies have assumed that crystal size is a significant contributor [60]. The width of the crystal peak (002) at half height is directly related to the crystal size, and the crystallite size's cellulose is about 4 to 7 nm in most references [61].

The crystallite size of cellulose-based OPEFB ranges from 1.36 to 3.42 nm, and cellulose has a small crystallite size. Crystalline cellulose is imperfect; thus, most cellulose structures are less regular and amorphous. Changes in crystallite size have not been fully correlated to the bleaching factor under acidic conditions.

According to Popescu et al. [56], the cellulose structure is more complicated than indicated by the two-phase (crystalline and amorphous) model. The amount of paracrystalline cellulose (33.1%) was almost identical to the amount of crystal structure (31.8%) in cotton cellulose. The existence of a transition region between the crystalline and amorphous structures makes it more challenging to interpret the crystallinity of cellulose. Likewise, if the amorphous structure is closed inside the crystallite structure, it will be difficult to react with the amorphous component, so changes in crystallinity will be challenging to predict.

### 3.5. Scanning Electron Microscopy

Morphological changes appear on the surface where, in OPEFB fiber, the surface morphology still looks rough; compared to fibers that have undergone bleaching, the surface area is smoother. Morphological changes in the extracted cellulose were smoother in the C3T2, C3.22T1, and C4.5T3 treatments. This smoother surface correlated with cellulose's physical properties, which were softer than the fiber after bleaching.

The bleaching process causes this subtle morphological change under acidic conditions, and alkaline delignification reduces the number of impurities, especially hemicellulose and lignin. Hemicellulose and lignin are amorphous, but hemicellulose is hydrogen bonded to cellulose, so it will be easier to remove than covalently bonded lignin.

**Figure 7:**
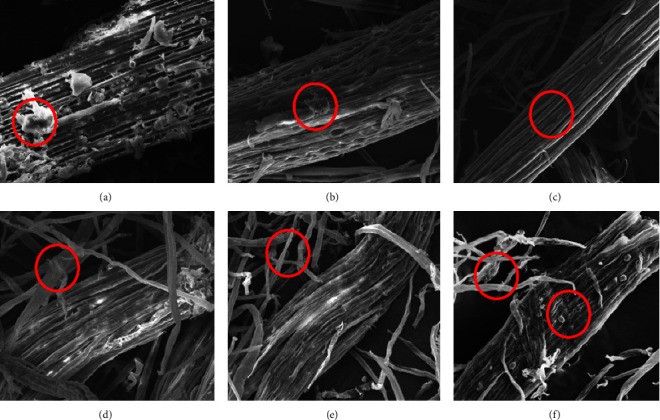
Morphology of OPEFB and cellulose fibers using 1000x magnification SEM, 100 mm size: (a) OPEFB fiber; (b) OPEFB bleached fiber; (c) OPEFB cellulose of NaClO_2_ 1.5% 3 h; (d) OPEFB cellulose of NaClO_2_ 3% 2 h; (e) OPEFB cellulose of NaClO_2_ 3.22% 1 h; and (f) OPEFB cellulose of NaClO_2_ 4.5% 3 h.

The morphological differences of cellulose in the C1.5T3, C3T2, and C4.5T3 treatments indicated that the C3T2 and C4.5T3 treatments had been able to open the cellulose structure.  Figure 7 shows the stretching of the structure's fibrils, which can later affect the degree of crystallinity of cellulose. Cellulose at optimum conditions C3.22T1 showed a morphology similar to C3T2. The C4.5T3 treatment showed a high concentration of NaClO_2_, and prolonged bleaching in acidic conditions had a more significant grinding effect on the cellulose structure; this would correlate with the lower yield amount in the treatment.

Based on Figure 7, the treatment of C3T2, C3.22T1, and C4.5T3 resulted in an increasingly intensive fibril separation. There was decreased wax, hemicellulose, and lignin structure; a smooth and scar-like surface supported this. According to Nazir et al. [63, 64], a scar-like surface is due to the removal of inorganic materials such as silica left behind by carbon and oxygen.

The study applied the whole fiber of OPEFB, where the hard part at the end of the stalk was not separated and is relatively more stubborn to pretreatment than the long fiber of the EFB structure. Reneta Nafu et al. [65] showed that more extensive degradation and intensive fibril opening occurred in OPEFB stem fibers with a softer structure than stalk fibers.

## 4. Conclusions

The bleaching process under acidic conditions is effective for improving the delignification performance to obtain high cellulose. Based on the response surface method, the results of the optimization of bleaching under acidic conditions using NaClO_2_ at pH 4–4.5 obtained the optimum bleaching conditions at a concentration of 3.22% NaClO_2_ for 1 hour and continued with the delignification of NaOH. Under these conditions, the purity of cellulose was 82.96% ± 2.53, hemicellulose 9.27% ± 2.28, and lignin 1.68% ± 0.58. High *α*-cellulose was needed in MCC production as a mechanical strength function. *α*-Cellulose in C3.22T1 treatment ranged from 83.02% to 94.00%. XRD test results showed that under these conditions, the samples had a crystallinity index of 70.55% and a crystallite size of 2.35 nm. Based on the SEM test, the morphology on the surface of the cellulose showed that the bleaching treatment under acidic conditions helped remove hemicellulose impurities, lignin, and inorganic materials, as well as a more intensive opening of cellulose fibrils. Cellulose from OPEFB has great potential in terms of quality and quantity, so the oil palm industry needs to use it in an integrated manner to become a high-value industrial product. The oil palm industry not only dumps OPEFB waste onto land as fertilizer but also converts OPEFB cellulose into microcrystalline cellulose, which is useful as a filler for various products or other cellulose derivative products.

## Figures and Tables

**Table 1:** Optimizing factors for bleaching on OPEFB.

Factor	Name	Minimum	Maximum	Coded low (−1)	Coded high (+1)	Mean (0)
*X *_1_	Bleaching time (h)	0.59	3.41	1.00	3.00	2.00
*X *_2_	Concentration of NaClO_2_ (%)	0.88	5.12	1.50	4.50	3.00

**Table 2:** Experimental design for optimizing cellulose isolation from OPEFB.

Std	Run	Factor 1	Factor 2
		*X *_1_: bleaching time (h)	*X *_2_: concentration of NaClO_2_ (%)
9	1	2	3
6	2	3.41	3
10	3	2	3
4	4	3	4.50
8	5	2	5.12
5	6	0.59	3
12	7	2	3
3	8	1	4.50
1	9	1	1.50
7	10	2	0.88
13	11	2	3
11	12	2	3
2	13	3	1.5

**Table 3:** Content of water-extractive material, hemicellulose, cellulose, holocellulose, and lignin in OPEFB cellulose.

Treatments	Water extractive material (%)	Hemicellulose (%)	Cellulose (%)	Holocellulose (%)	Lignin (%)
C3T2	5.48	11.30	80.43	91.73	2.78
C3T0.59	6.20	7.32	84.79	92.11	1.69
C3T2	7.12	9.97	81.20	91.17	1.71
C3T3.41	8.24	14.20	75.85	90.05	1.70
C3T2	6.46	7.58	83.71	91.29	2.25
C4.5T1	7.41	10.83	80.06	90.89	1.71
C1.5T1	7.48	7.76	79.50	87.26	5.26
C5.12T2	8.20	11.67	78.86	88.53	1.26
C0.88T2	6.76	15.77	68.17	83.94	9.30
C4.5T3	8.45	8.17	81.69	89.86	1.69
C1.5T3	8.19	11.58	74.01	85.59	6.21
C3T2	9.40	10.54	77.78	88.32	2.28
C3T2	8.78	9.92	78.47	88.39	2.83

**Table 4:** Fit summary.

Source	Sequential *p* value	Lack of fit *p* value	Adjusted *R*^2^	Predicted *R*^2^	

*Water extractive material*					
**Linear**	**0.2921**	**0.9948**	**0.0618**	**−0.0051**	**Suggested**
2FI	0.8887	0.9869	−0.0401	−0.1636	
Quadratic	0.9552	0.9269	−0.3198	−0.6253	
Cubic	0.8789	0.6666	−0.7548	−2.4760	Aliased

*Hemicellulose*					
Linear	0.2309	0.0902	0.1049	−0.5146	
**2FI**	**0.1895**	**0.0998**	**0.1874**	**−0.6467**	**Suggested**
Quadratic	0.3502	0.0855	0.2258	−1.6543	
Cubic	0.3142	0.0537	0.3179	−10.9214	Aliased

*Cellulose*					
Linear	0.0480	0.1523	0.3462	−0.0484	
2FI	0.3343	0.1437	0.3488	−0.0409	
**Quadratic**	**0.0439**	**0.3721**	**0.6572**	**0.1251**	**Suggested**
Cubic	0.3449	0.3180	0.6865	−1.2036	Aliased

*Lignin*					
Linear	0.0024	0.0100	0.6418	0.3982	
2FI	0.7516	0.0075	0.6067	0.2999	
**Quadratic**	**0.0006**	**0.2024**	**0.9390**	**0.8163**	**Suggested**
Cubic	0.0987	0.5159	0.9662	0.8790	Aliased

Bold type indicates the suggested model for response.

**Table 5:** Lack of fit value.

Source	*F*-value	*p* value	Sign
*1. Water extractive material*			
*X *_1_-bleaching time	2.27	0.1629	
*X *_2_-concentration of NaClO_2_	0.5211	0.4869	
Lack of fit	0.0846	0.9948	Not significant

*2. Hemicellulose*			
*X *_1_-bleaching time	2.85	0.1258	
*X *_2_-concentration of NaClO_2_	0.9052	0.3662	
*X *_1_ * X *_2_	2.01	0.1895	
Lack of fit	4.06	0.0998	Not significant

*3. Cellulose*			
*X *_1_-bleaching time	5.30	0.0548	
*X *_2_-concentration of NaClO_2_	10.63	0.0139	
*X *_1_ * X *_2_	1.98	0.2025	
*X*′_1_^2^	0.2453	0.6355	
*X*′_2_^2^	9.28	0.0187	
Lack of fit	1.37	0.3721	Not significant

*4. Lignin*			
*X *_1_-bleaching time	0.3304	0.5834	
*X *_2_-concentration of NaClO_2_	137.60	<0.0001	
*X *_1_ * X *_2_	0.6863	0.4348	
*X*′_1_^2^	1.57	0.2499	
*X*′_2_^2^	46.33	0.0003	
Lack of fit	2.46	0.2024	Not significant

**Table 6:** Constraint data of optimization.

Name	Goal	Lower limit	Upper limit	Lower weight	Upper weight	Importance
*X *_1_: bleaching time	Is in range	1	3	1	1	3
*X *_2_: concentration of NaClO_2_	Is in range	1.5	4.5	1	1	3
Water extractive material	Minimize	5.483	9.402	1	1	3
Hemicellulose	Minimize	7.324	15.775	1	1	3
Cellulose	Maximize	68.169	84.788	1	1	3
Lignin	Minimize	1.262	9.296	1	1	3

**Table 7:** Confirmation of results at the optimization point on the response surface methodology.

Response	Predicted mean	95% PI low	95% PI high
Water-extractive material	7.013 ± 1.089	4.348	9.678
Hemicellulose	9.273 ± 2.283	3.590	14.955
Cellulose	82.959 ± 2.533	76.200	89.718
Lignin	1.682 ± 0.586	0.119	3.246

**Table 8:** Data on the crystallinity index and crystallite size of cellulose extracted from OPEFB fiber.

Treatments	2*θ* (*I*_002_)	2*θ* (*I*_*am*_)	Crystallinity index (%)	Crystallite size (nm)
C3T2	23.21	16.40	85.78	1.95
C3T0.59	23.06	16.57	86.56	1.36
C3T3.41	23.11	17.05	81.36	2.54
C3T2	23.27	16.62	84.39	2.47
C4.5T1	23.58	17.10	79.39	2.80
C1.5T1	23.09	16.16	80.19	2.16
C5.12T2	23.21	16.99	89.15	2.12
C0.88T2	23.27	16.35	74.75	2.49
C4.5T3	22.57	15.38	72.64	3.42
C1.5T3	22.67	15.70	60.88	3.19
C3.22T1	23.09	16.35	70.55	2.35

C: concentration of NaClO_2_ (%); T = bleaching time (h).

## Data Availability

The data used to support the findings of this study are included within this article, and necessary data can be obtained from the corresponding author upon request.
